# Supplementary material for: Engineering cell morphology by CRISPR interference in Acinetobacter baylyi ADP1
Source: Microb Biotechnol. 2022 Aug 25;15(11):2800–18. doi: 10.1111/1751-7915.14133 (PMC9618324; doi:10.1111/1751-7915.14133)
Supplement: Supplementary file 1 — Appendix S1 [file MBT2-15-2800-s003.docx]

**Engineering cell morphology by CRISPR interference in *Acinetobacter baylyi* ADP1**

Jin Luo^1^*, Elena Efimova^1^, Daniel Christoph Volke^2^, Ville Santala^1^, Suvi Santala^1^
^1^Faculty of Engineering and Natural Sciences, Hervanta campus, Tampere University, Korkeakoulunkatu 8, Tampere, 33720, Finland

^2^ The Novo Nordisk Foundation Center for Biosustainability, Technical University of Denmark, Kemitorvet 220, 2800 Kgs. Lyngby, Denmark

*Corresponding author

Email addresses:

Jin Luo: [jin.luo@tuni.fi](mailto:jin.luo@tuni.fi)
Elena Efimova: elena.efimova@tuni.fi
Daniel Christoph Volke: chdavo@biosustain.dtu.dk
Ville Santala: [ville.santala@tuni.fi](mailto:ville.santala@tuni.fi)
Suvi Santala: [suvi.santala@tuni.fi](mailto:suvi.santala@tuni.fi)

**Supplemental Materials**


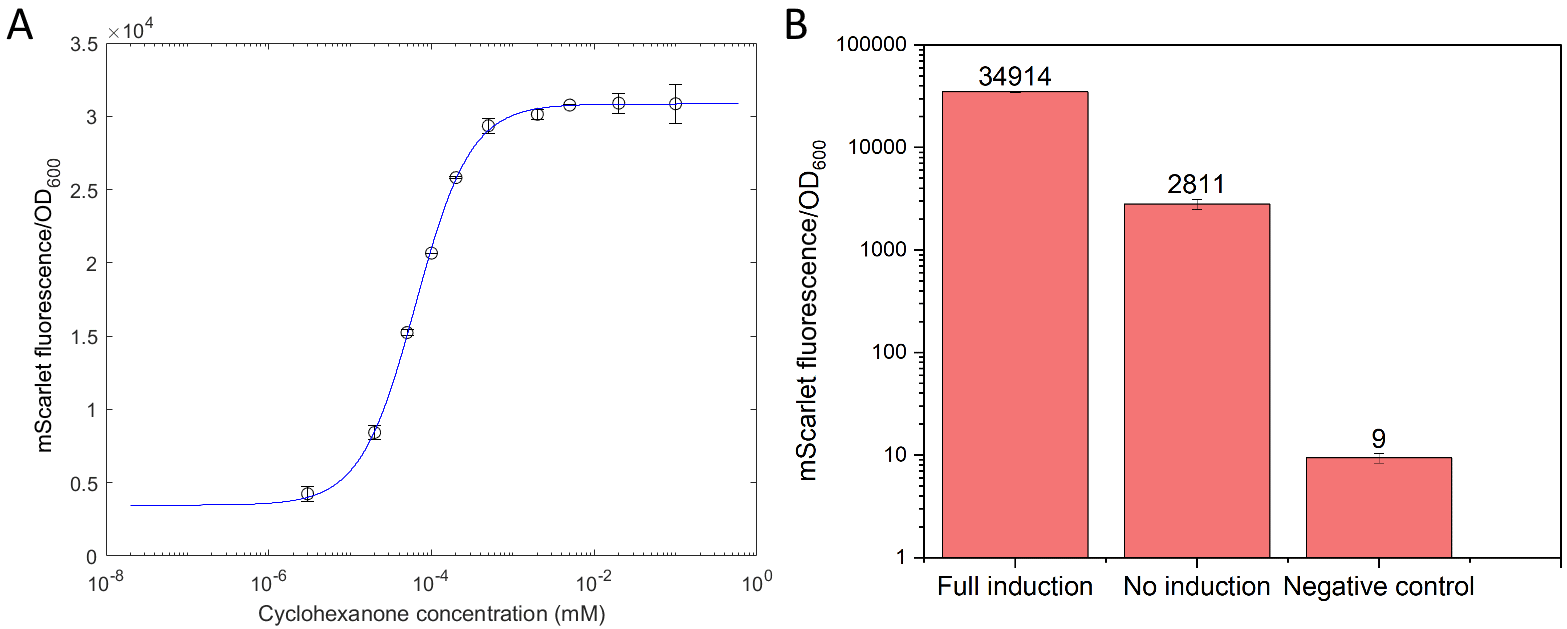


**Figure S1.** Characterization of the cyclohexanone-controlled induction system. (A) The transfer function of the cyclohexanone-inducible promoter. The transfer function shows how the activity of the cyclohexane-inducible promoter (expressed as mScarlet fluorescence/OD_600_) changes with cyclohexane concentration. The cyclohexanone induction system was integrated at the prophage site of the genome (Murin *et al.*, 2012; Lehtinen *et al.*, 2017), and the expression of *mScarlet* was driven by the RBS BBa_0034 (iGEM Part Registry). The blue line shows the fitting to the data points using the model shown in equation 3 (see Supplemental Note). (B) An independent experiment was performed to evaluate the leakiness of the system. The strain was grown in LB media with 5 µM cyclohexanone for full induction and without cyclohexanone. Wild-type ADP1 was used as the negative control. All the measurements were performed after 25 h of cultivation in LB media. Data represent average values ± standard deviations of two independent biological experiments.


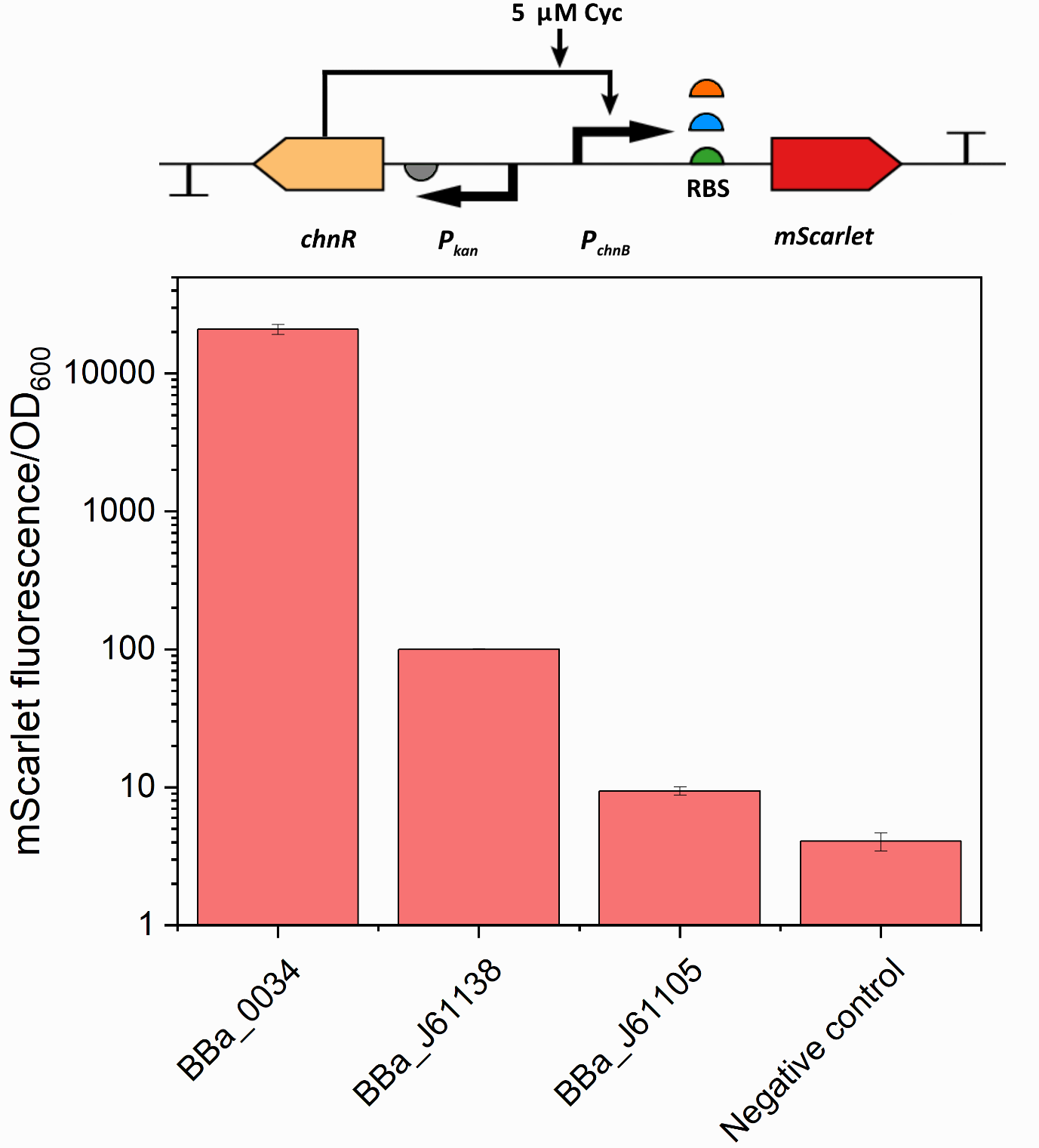


**Figure S2.** Comparison between the strength of three ribosome binding sites (RBSs) in ADP1. The top panel shows the architecture of the reporter construct. The three RBSs, BBa_0034, BBa_J61138, and BBa_J61105, are upstream of *mScarlet*, which is under the control of the cyclohexanone-inducible promoter. The *chnR* regulator is constitutively expressed by the *P_kan_* promoter (promoter of the kanamycin resistance gene). The cassette was integrated into the prophage site of the genome. All strains were grown in LB media containing 5 µM cyclohexanone (Cyc) at 30 ℃. Fluorescence data at 24 h was shown. Wild-type ADP1 was used as the control. Data represent average values ± standard deviations of two independent biological experiments.


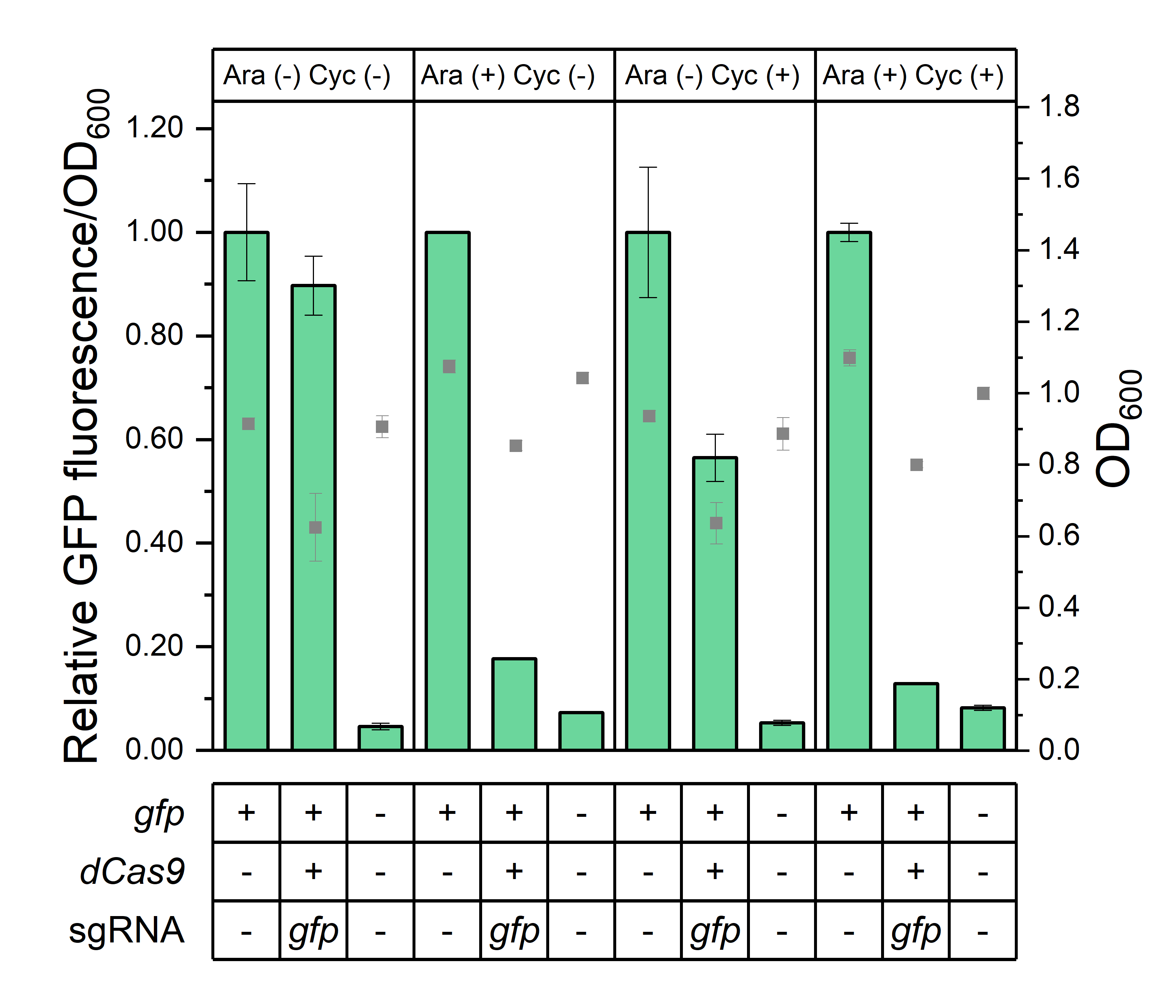


**Figure S3.** Analysis of the CRISPRi system with the strong RBS BBa_0034 driving the expression of *dCas9*. Cells were grown in LB media at 30 ℃ in the presence (+) or absence (-) of arabinose (1% w/v) and cyclohexanone (0.2 µM). For each condition, the data at 26 h is shown as the fluorescence/OD_600_ relative to the values from the GFP reporter strain without sgRNA and *dCas9*. The table indicates either the presence (+) or the absence (-) of the genome-integrated *gfp*, *dCas9*, and the sgRNA-expressing plasmid. The square symbols in grew show the OD_600_ when the fluorescence measurement was performed. Data represent average values ± standard deviations of two independent biological experiments.

**
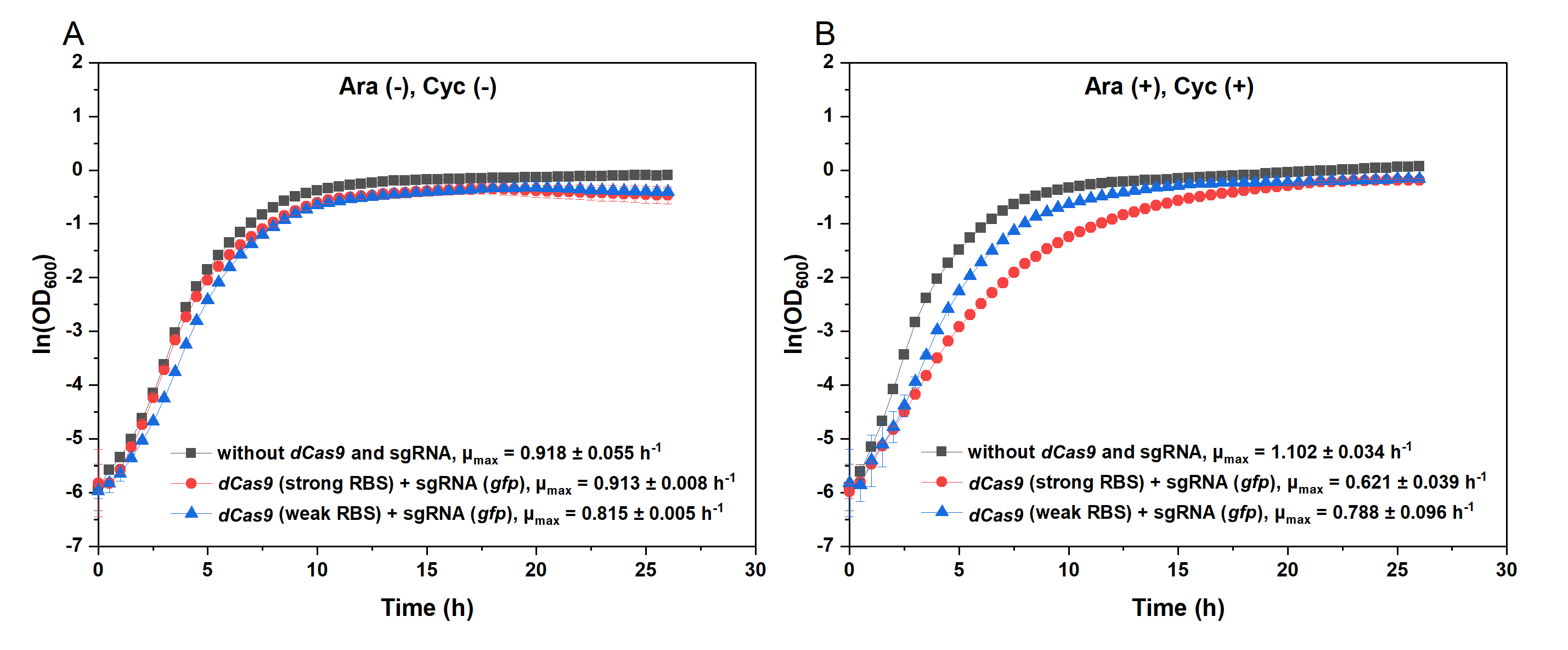
**

**Figure S4.** The effect of *dCas9* expression level on growth rate. The growth in non-inducing (A) and inducing (B) conditions are shown. The two strains ASA515 and ASA516 contain different RBSs preceding the *dCas9* gene: either the strong BBa_0034 or the weak BBa_J61138. The sgRNA targets the constitutively expressed *gfp*. The reporter strain ASA514 without the CRISPRi machinery was used as a control. Cells were cultivated in LB media at 30 ℃. For induction, 1% (w/v) arabinose (Ara) and 5 µM cyclohexanone (Cyc) were added in the beginning. The maximal specific growth rate (µ_max_) was calculated by taking the slope for the linear part of the logarithmic OD_600_ (R^2^ >0.99). Data represent average values ± standard deviations of two independent biological experiments.


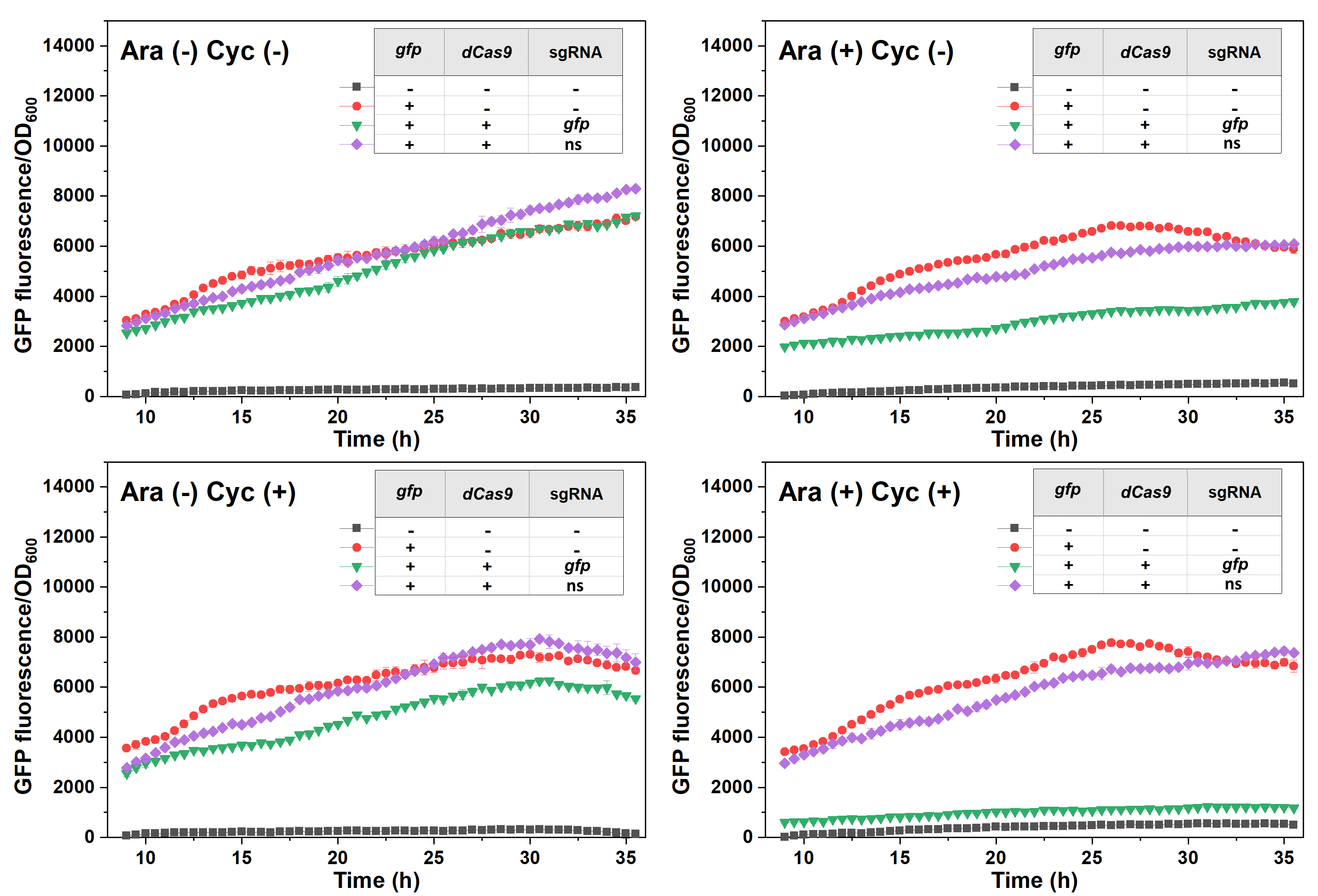


**Figure S5.** Change of GFP fluorescence/OD_600_ during cultivation for each ADP1 strain under different induction states. Cells were grown in LB media and cultivated at 30 ℃. For induction, 1% (w/v) arabinose (Ara) and/or 5 µM cyclohexanone (Cyc) were supplemented. The non-specific (ns) sgRNA was used as a control. Data represent average values ± standard deviations of two independent biological experiments.


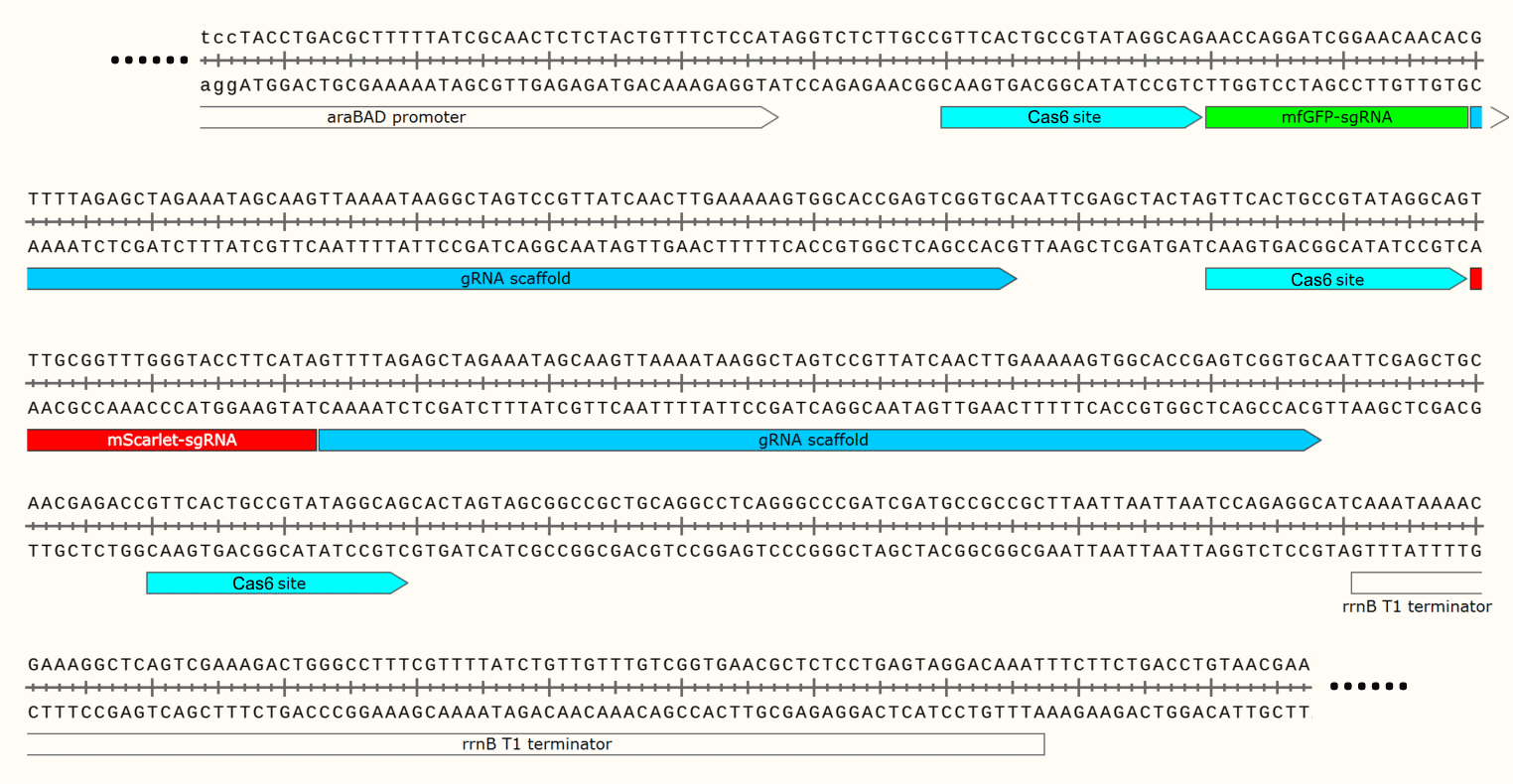


**Figure S6.** DNA sequence of the guide RNA array containing the *gfp*-targeting sgRNA and the *mScarlet*-targeting sgRNA.


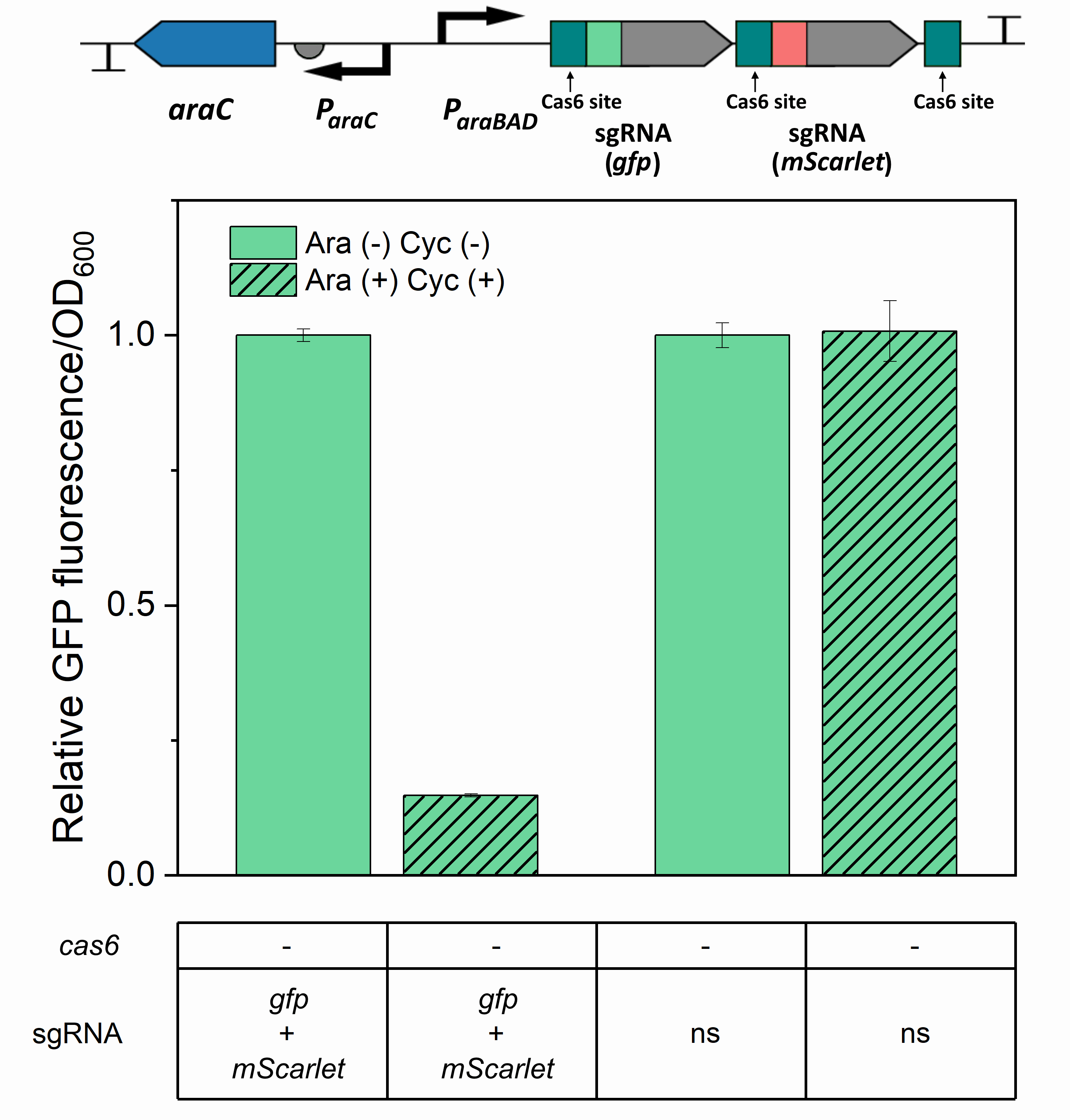


**Figure S7.** Validation of GFP repression by expression of the two-sgRNA array without *cas6*. To further validate that the *gfp*-targeting sgRNA, which was placed before the *mScarlet*-targeting sgRNA in the two-sgRNA array (as shown in the top panel), was still functional without *cas6*, the cassette (integrated into the pBAV plasmid) was introduced into the GFP single reporter strain containing *dCas9*. The GFP reporter strain containing *dCas9* and a non-specific (ns) sgRNA was used as control. Cells were grown in buffered LB containing 0.4% (w/v) glucose and cultivated at 30 ℃. For induction, 1% (w/v) arabinose (Ara) and 5 µM cyclohexanone (Cyc) were supplemented. Samples were taken for fluorescence measurement at 20 h. The fluorescence values for each strain were normalized to the values measured for this strain without induction. Data represent average values ± standard deviations of two independent biological experiments.


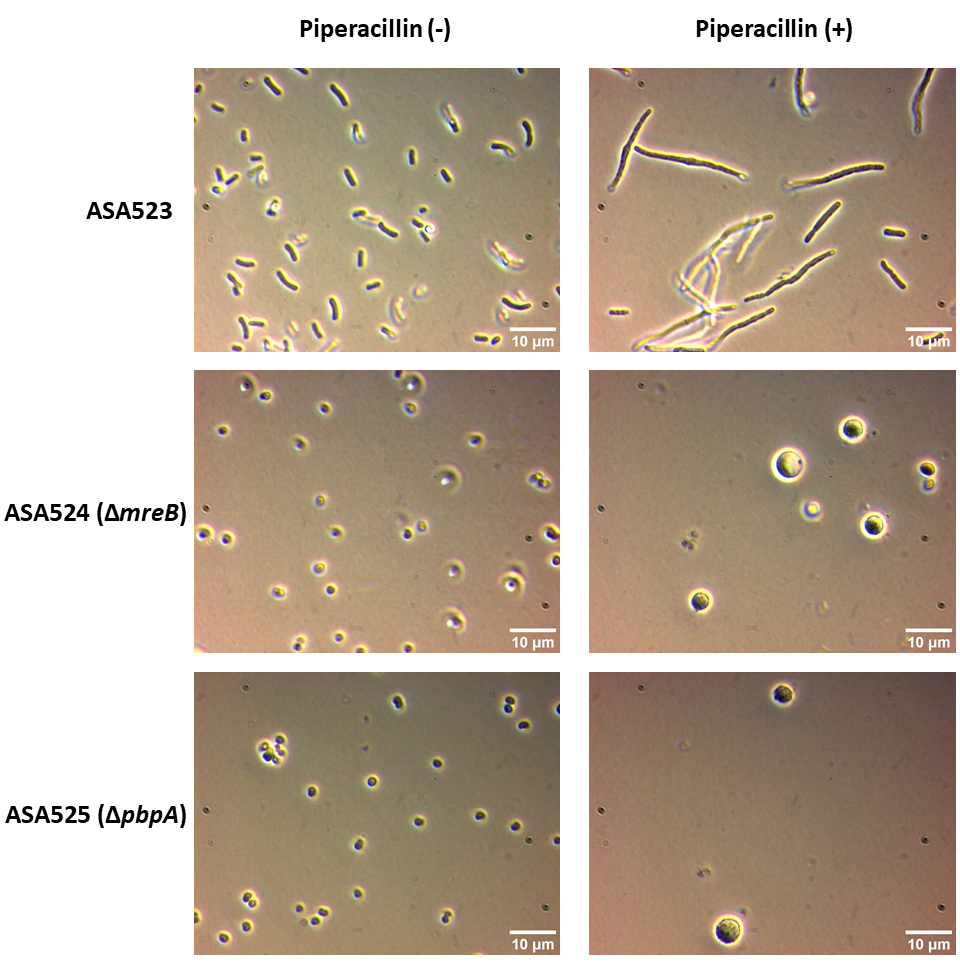


**Figure S8.** Cells treated with piperacillin were enlarged. Cells were cultivated in MA/9 media supplemented with 200 mM glucose at 25 ℃ for 20 h. Piperacillin with a final concentration of 12 µg/mL was used for the treatment. The strain ASA523 contained a copy of acr1 under the control of the constitutive promoter T5. ASA524 and ASA525 were derived from ASA523 by deleting the rod shape-related genes mreB and pbpA respectively.





**Figure S9.** Cultivation of ASA531 (*aceA* deletion + *acr1* overexpression + CRISPRi targeting *ftsZ*) in glucose and acetate. Cells were grown in mineral salts media containing 0.2% casamino acids, 5 mM glucose, and 60 mM acetate, and cultivated at 30 ℃. Glucose (5 mM ) and acetate (60 mM) were fed again at 10 h and 21 h. Two parallel cultivations were performed, and inducers (1% (w/v) arabinose and 50 µM cyclohexanone) were added when the OD reached ~0.5 (early induction) and ~1.5 (late induction) respectively. The OD (top panel), glucose concentration (middle panel), and acetate concentration (bottom panel) were measured at 0, 10, 21, and 34 h. Data represent average values ± standard deviations of two independent biological experiments.

**
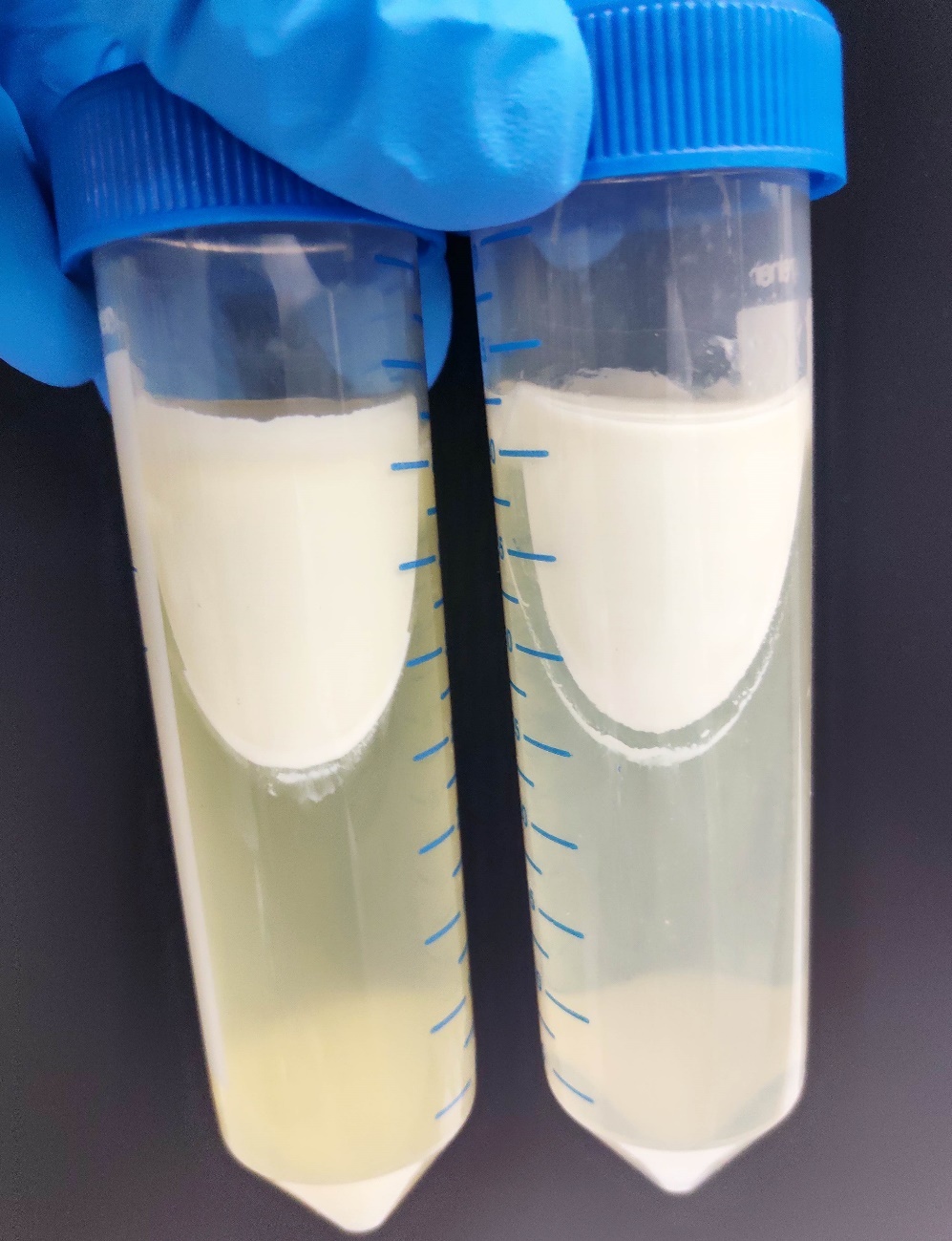
**

**Figure S10.** Cultures after centrifugation for harvesting. Part of the culture was floating or suspended in the media after centrifugation (16743 *g* for 2.5 h) when fatty acids were used as the substrate for the cultivation.


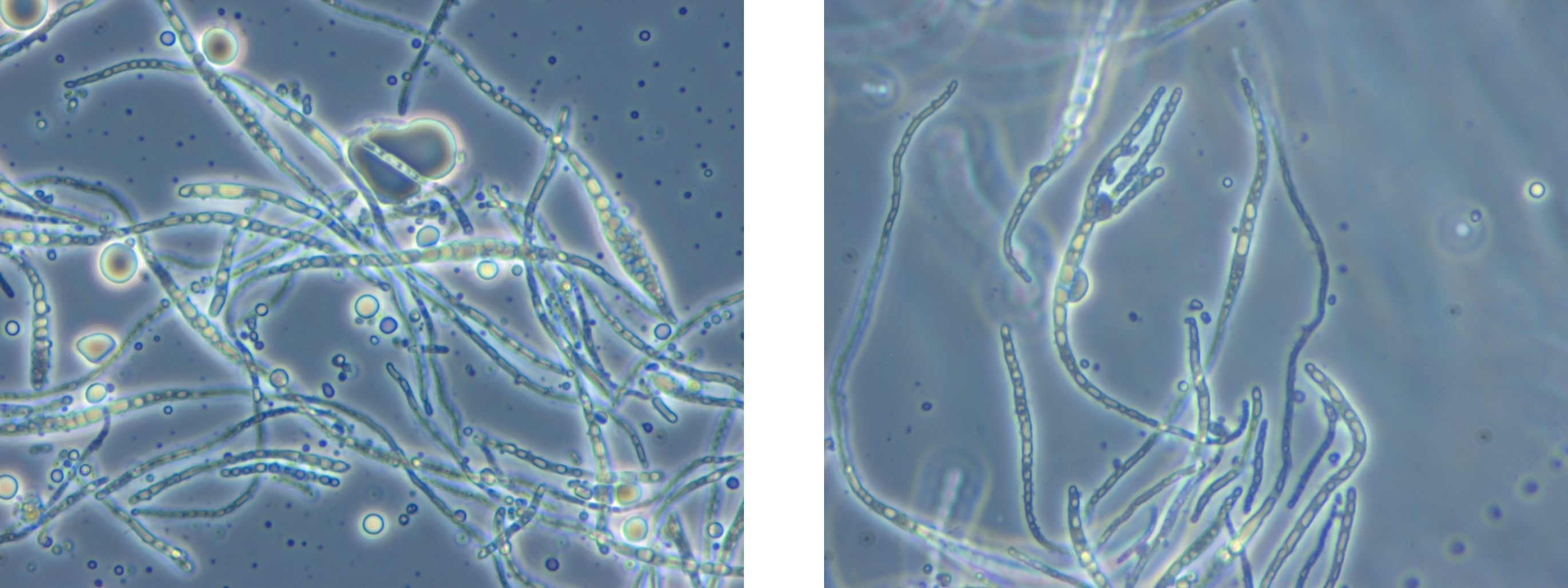


**Figure S11.** Microscopic observation of the elongated cells in the low-density fraction (left) and the pellet (right) after centrifugation. The strain ASA531 was cultivated by co-feeding of glucose and fatty acids for 36 h and was induced for elongation at early stage (12 h). The microscopic photo on the right is the same as the one from Figure 7E and was presented here for comparison.

**Supplemental Note**

**Mathematical analysis for the cyclohexanone induction system**

The regulator ChnR was reported to be an activator (Iwaki *et al.*, 1999; Steigedal and Valla, 2008), in response to cyclohexanone. The binding of cyclohexanone (Cyc) to ChnR is described with equation 1,

$$\text{F}\text{ChnR:Cyc}=\frac{\left[ \mathrm{Cyc} \right]^{n}}{K_{d}^{n}+\left[ \mathrm{Cyc} \right]^{n}} \left( 1 \right)$$

where F_chnR:Cyc_ is the fraction of ChnR bound to cyclohexanone, [Cyc] is cyclohexanone concentration, K_d_ is the dissociation constant, and n is the cooperativity. The activity of the promoter is described with equation 2,

$$\text{P}\text{chnB}=P_{\mathrm{chnB}}^{\max}*\frac{\text{K}\text{1}\text{+K}\text{2}*\text{F}\text{ChnR:Cyc}}{1+\text{K}\text{1}\text{+K}\text{2}*\text{F}\text{ChnR:Cyc}} \left( 2 \right)$$

where P_chnB_ is the activity of the cyclohexanone-inducible promoter, Pis the maximal promoter activity. K_1_and K_2_ are the terms of two states of the promoter (Figure S11). As the promoter activity can be represented by the fluorescence signal, the fluorescence signal can be described using a similar equation below,

$$\left[ fluorescence/OD \right]=\left[ fluorescence/OD \right]_{\max}*\frac{\text{K}\text{1}\text{+K}\text{2}*\text{F}\text{ChnR:Cyc}}{1+\text{K}\text{1}\text{+K}\text{2}*\text{F}\text{ChnR:Cyc}} (3)$$

Where [fluorescence/OD] is the measured fluorescence per OD, [fluorescence/OD]_max_ is the maximal fluorescence signal. The parameters for fitting the model to the experimental data are shown in Table S1.


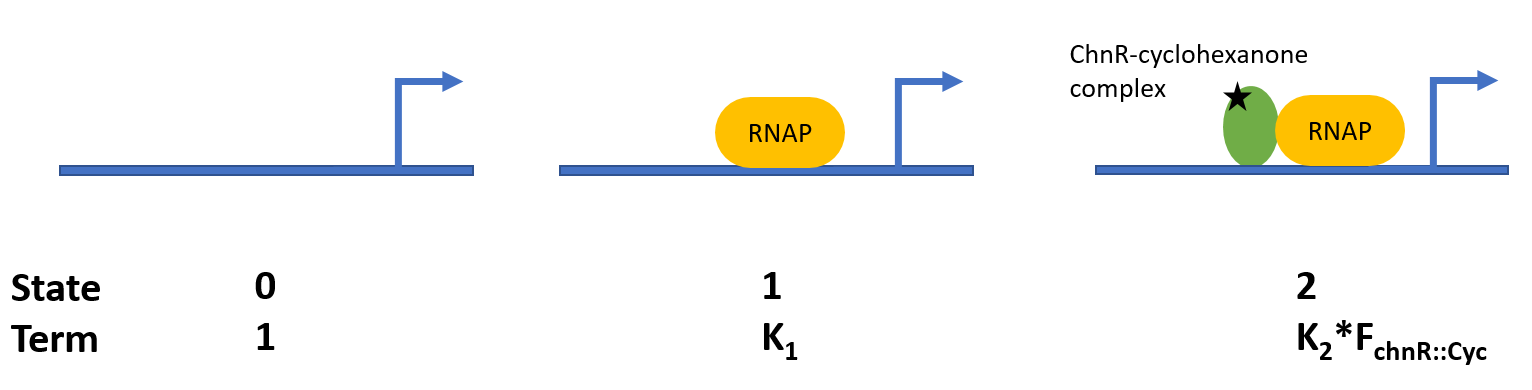
**Figure S11.** A model assuming three major states of the cyclohexanone-inducible promoter. In the first state, the promoter is not occupied. In the second state, the promoter binds to RNA polymerase. In the third state, the promoter is activated, binding to the ChnR-cyclohexanone complex and RNA polymerase. The amounts of RNA polymerase and ChnR are assumed to be constant.

**Table S1.** Parameters for data fitting.

| [fluorescence/OD]_max_ | K_1_ | K_2_ | K_d_ | n |
| --- | --- | --- | --- | --- |
| 31718 | 0.12 | 35.02 | 0.00095 | 1.28 |

**References**

Iwaki, H., Hasegawa, Y., Teraoka, M., Tokuyama, T., Bergeron, H., and Lau, P.C.K. (1999) Identification of a Transcriptional Activator (ChnR) and a 6-Oxohexanoate Dehydrogenase (ChnE) in the Cyclohexanol Catabolic Pathway in Acinetobacter sp. Strain NCIMB 9871 and Localization of the Genes That Encode Them. *Appl Environ Microbiol* **65**: 5158–5162.

Lehtinen, T., Santala, V., and Santala, S. (2017) Twin-layer biosensor for real-time monitoring of alkane metabolism. *FEMS Microbiol Lett* **364**: 1–7.

Murin, C.D., Segal, K., Bryksin, A., and Matsumura, I. (2012) Expression Vectors for Acinetobacter baylyi ADP1. *Appl Environ Microbiol* **78**: 280–283.

Steigedal, M. and Valla, S. (2008) The Acinetobacter sp. chnB promoter together with its cognate positive regulator ChnR is an attractive new candidate for metabolic engineering applications in bacteria. *Metab Eng* **10**: 121–129.
